# Influence of Amorphous Boron Grain Size, High Isostatic Pressure, Annealing Temperature, and Filling Density of Unreacted Material on Structure, Critical Parameters, *n*-Value, and Engineering Critical Current Density in MgB_2_ Wires

**DOI:** 10.3390/ma14133600

**Published:** 2021-06-28

**Authors:** Daniel Gajda, Andrzej J. Zaleski, Andrzej Morawski, Małgorzata Małecka, Mustafa Akdoğan, Firat Karaboğa, Doğan Avcı, Hakan Yetiş, Ibrahim Belenli, Tomasz Czujko

**Affiliations:** 1 Institute of Low Temperature and Structure Research PAS, Okolna 2, 50-422 Wroclaw, Poland; a.zaleski@intibs.pl (A.J.Z.); m.malecka@int.pan.wroc.pl (M.M.); 2 Institute of High Pressure Physics PAS, Sokolowska 29/37, 01-142 Warsaw, Poland; amor@unipress.waw.pl; 3 Department of Physics, Bolu Abant Izzet Baysal University, 14280 Bolu, Turkey; akdogan_m@ibu.edu.tr (M.A.); davci.0209@gmail.com (D.A.); hknyetis@gmail.com (H.Y.); belenli_i@ibu.edu.tr (I.B.); 4 Mehmet Tanrıkulu Health Services Vocational School, Bolu Abant Izzet Baysal University, 14030 Bolu, Turkey; karabogafirat@ibu.edu.tr; 5 Institute of Materials Science and Engineering, Military University of Technology, Kaliskiego 2, 00-908 Warsaw, Poland

**Keywords:** MgB_2_ superconducting wires, boron grain size, density of unreacted material, high isostatic pressure, critical current density

## Abstract

Our results show that a lower density of unreacted Mg + B material during an Mg solid-state synthesis reaction leads to a significant reduction in the quantity of the superconducting phase and lowers the homogeneity of the superconducting material. It also significantly reduces the irreversible magnetic field (B_irr_), critical temperature (T_c_), upper magnetic field (B_c2_), engineered critical current density (J_ec_), and *n*-value, despite high isostatic pressure (HIP) treatment and the use of nanoboron in the sample. Our measurements show that samples with large boron grains with an 8% higher density of unreacted Mg + B material allow better critical parameters to be achieved. Studies have shown that the density of unreacted material has little effect on B_irr_, T_c_, B_c2_, J_ec_, and the *n*-value for an Mg liquid-state synthesis reaction. The results show that the critical parameters during an Mg liquid-state synthesis reaction depend mainly on grain size. Nanoboron grains allow for the highest B_irr_, T_c_, B_c2_, J_ec_, and *n*-values. Scanning electron microscopy (SEM) images taken from the longitudinal sections of the wires show that the samples annealed under low isostatic pressure have a highly heterogeneous structure. High isostatic pressure heat treatment greatly improves the homogeneity of MgB_2_.

## 1. Introduction

Currently, MgB_2_ wires are the most promising material for superconducting coil applications due to their inexpensive components and ability to be cooled in liquid hydrogen, or by a cryocooler. The application of superconducting wires is dependent on several factors, e.g., engineering critical current density (J_ec_), irreversible magnetic field (B_irr_), critical temperature (T_c_), *n*-value (the log-log slope of the voltage vs. electrical current curves during the transition of the superconducting material from a superconducting state to a normal state), and resistance in the normal state (R_n_). These parameters depend on the grain size and purity of magnesium and boron, heat treatment temperature, isostatic pressure, admixture, and wire sheath. Research has shown that nanoamorphous boron allows more connections between grains to be obtained, improves critical current density (J_c_), and accelerates the synthesis reaction [1,2,3,4,5,6,7]. However, large grains of amorphous boron (1 µm) result in a smaller number of connections between the grains, reduce the critical current density, and slow down the synthesis reaction [1,6]. However, nanoamorphous boron is much more expensive than large-grain amorphous boron, leading to a higher price for MgB_2_ wires. In addition, J_c_ also depends on the purity and type of boron [2,4]. Research has shown that high purity boron can significantly increase J_c_, B_irr_, and T_c_. Moreover, experiments have shown that amorphous boron provides higher J_c_ and B_irr_ values than crystalline boron [1]. The research presented by Kim et al. indicates that nanocrystalline boron has a greater number of defects (e.g., dislocations, stresses) than large crystalline boron [1]. Additionally, these studies show that a greater number of structural defects in boron can accelerate the formation of the MgB_2_ superconducting phase.

Thermal treatment under high isostatic pressure (HIP) increases the density and homogeneity of MgB_2_ material, leads to more connections between grains, reduces the size of voids, forms structural defects (e.g., dislocations and strains), and creates smaller MgB_2_ grains [6,8,9,10,11]. Additionally, studies show that isostatic pressure increases the melting point of Mg [11,12], allowing synthesis reactions to be carried out in the solid-state of Mg. Moreover, thermal treatment under high isostatic pressure enhances the irreversible magnetic field and critical current density [6,13,14].

Currently, synthesis reactions for MgB_2_ materials are performed by two methods: Mg solid-state and Mg liquid-state. The reaction in the solid-state of Mg forms small MgB_2_ grains, smaller voids, and a greater number of intergrain connections [15,16,17,18]. The disadvantage of this reaction is the residual unreacted Mg and B, which can be eliminated by applying a long annealing time [11]. Synthesis reactions in the liquid state of Mg can be conducted in a short time, allowing one to obtain a large number of superconducting phases after a short thermal treatment time. This type of reaction leads to the formation of large MgB_2_ grains, large voids, and fewer connections between grains, resulting in a degradation in critical current density [15,18].

A study of the MgB_2_ material showed that the density of the unreacted material (Mg + 2B) affects the critical parameters of MgB_2_ wires. Flükiger et al. [19], Hossain et al. [20], and Akdoğan et al. [21] showed that a higher density of unreacted material (Mg + 2B) increases critical current density in MgB_2_ wires. The core density of the unreacted (Mg + 2B) depends on the outer sheath material of the wires. Sheathing with a high hardness, e.g., Fe, results in a higher unreacted core density than sheathing with a lower hardness, e.g., Cu [22]. Tanaka et al. showed that a low mass density of unreacted Mg + 2B in the wire leads to a reduction in J_c_ for the superconducting coil [23]. Susner et al. indicated that both cold pressure and hot pressure processes increase the density of MgB_2_ material (after heat treatment) and improve the J_c_ of MgB_2_ wires [24].

The application of MgB_2_ wires depends mainly on two factors: *n*-value and J_ec_. The *n*-value describes the transition from a superconducting state to a normal state [25,26,27] and depends on intrinsic (e.g., connections between grains, grain microstructure, and pinning centers) and extrinsic (e.g., filament distribution and quantity) factors. Kim et al. noted that a wire with a high *n*-value is used for a magnet to reduce its resistive component. Additionally, research shows that a high-quality sample has a high *n*-value. Moreover, Kim et al. indicated that MgB_2_ wires with a highly uniform microstructure reduce the dissipation for magnet applications [25]. Research by Motaman et al. also indicates that a more homogeneous microstructure causes a larger *n*-value, and that the *n*-value mostly decreases due to the inhomogeneous parts of samples [26]. Kim et al. showed that a small *n*-value, which leads to thermal and electrical dissipation, is characteristic of MgB_2_ superconductors [25,26]. Motaman et al. suggested that structural defects (dislocations) lead to an increase in B_c2_ and J_c_ in high magnetic fields but reduce the *n*-value [26]. The results presented by Parizh et al. show that an *n*-value of 20 allows the use of only 50% of the potential critical current in superconducting wires and tapes [27]. Motaman et al. indicated that superconducting wires are used in magnets when the *n*-value is in the range of 50 to 100 [26]. The J_ec_ of the wires is used as a parameter in designing superconducting magnets. This value determines the critical current density (J_c_) for the cross-section of superconducting wire and tape (e.g., diffusion barrier, wire sheath, heat matrix, and superconducting material) [28,29]. Depending on the device in which the superconducting coils are used, the J_ec_ value is currently 20–50 A/mm^2^ for magnetic resonance imaging (MRI) magnets, 20–50 A/mm^2^ for large coils for detectors, and 100–200 A/mm^2^ for laboratory solenoids [28].

B_irr_ is dependent on high-field pinning centers that are created by structural defects, e.g., dislocations, stresses, strains, and substitutions of C for B [29,30,31,32]. MgB_2_ material has an advantage of having a high critical temperature of 39 K [33]. Thus, cooling of the MgB_2_ wires and tapes can be achieved using liquid hydrogen, cryocoolers, or solidified nitrogen, which significantly reduces the running costs of superconducting devices. Additionally, MgB_2_ material has a low resistivity in the normal state of 0.38 μΩcm [34]. This facilitates the design of protections against damage to the wires during the transition from a superconducting state to a normal state.

Our research aim was to show the effects of boron grain size, density of unreacted material, magnesium state (solid or liquid), and thermal treatment under high isostatic pressure on the formation of the MgB_2_ superconducting phase, the structure of the superconducting material, and critical parameters (B_irr_, B_c2_, T_c_, R_n_, and J_c_).

## 2. Materials and Methods

Monocore MgB_2_ wires with iron sheaths were produced using magnesium powder (purity: 99%—particle sizes: 100–200 mesh ~149–74 μm) (Abant Izzet Baysal University, Bolu, Turkey) nd amorphous boron powder (Pavezyum Advanced Chemicals, İstanbul, Turkey).

Samples A to E were made using amorphous boron of 1 µm particle size (Table 1). The samples from F to J were manufactured with amorphous boron mixtures: 50 wt %—amorphous boron of 1 µm particle size, and 50 wt %—nanoamorphous boron of 0.25 µm particle size (Table 1). Finally, the samples from K to O were fabricated with amorphous boron of 0.25 µm particle size.

The stoichiometric precursor Mg+2B powder was mixed by ball milling for 3 h using a Pascall Engineering 1600 VSA ball mill at 150 rpm and a ball-powder ratio of 4:1. The initial filling density of the filled powder for the samples from A to J was approximately 1.5 g/cm^3,^ and the filling density for the samples from K to O was approximately 1.38 g/cm^3^ (Table 1) [21]. A cold drawing technique, with some intermediate heat treatment steps, was used to produce wire samples with a final diameter of 1.00-mm. Details of the production methods are presented in [21]. All samples were heated in an argon atmosphere at temperatures ranging from 680 °C to 740 °C, and under isostatic pressures ranging from 0.1 MPa to 1.1 GPa for 40 min, as listed in Table 1 [35].

Measurements of B_irr_, T_c_, B_c2_, and R_n_ were made using a physical property measurement system (Physical Property Measurement System (PPMS), Model 7100, 15 Hz and AC current, I_AC_ = 100 mA, Quantum Design, San Diego, CA, USA). The critical parameters B_irr_, T_c_, and B_c2_ were identified for the criteria of 10%, 50%, and 90% resistance in the normal state [18]. The resistance in the normal state (R_n_) for B = 0 T was determined at 40 K. However, magnetoresistance was determined in the range of the magnetic field from 0 T to 14 T. Transport critical current measurements were made using a measurement system with a bitter magnet up to 14 T at a temperature of 4.2 K. The critical current (I_c_) was determined based on the 1 µm/cm criterion [29]. J_ec_ was calculated from the relationship J_ec_ = I_c_/S (S—wire cross-section) [29]. The *n*-values were determined from characteristic transport measurements [27]. The J_ec_ and *n*-values for all samples were tested at the temperature of liquid helium (4.2 K).

The structure and composition studies were performed by using a scanning electron microscope (FEI Nova Nano SEM 230, Hillsboro, OR, USA) and Ultra Plus Scanning Electron Microscope (Zeiss, Oberkochen, Germany).

## 3. Results and Discussion

### 3.1. Analysis of the Structure of MgB_2_ Material

**Figure 1:**
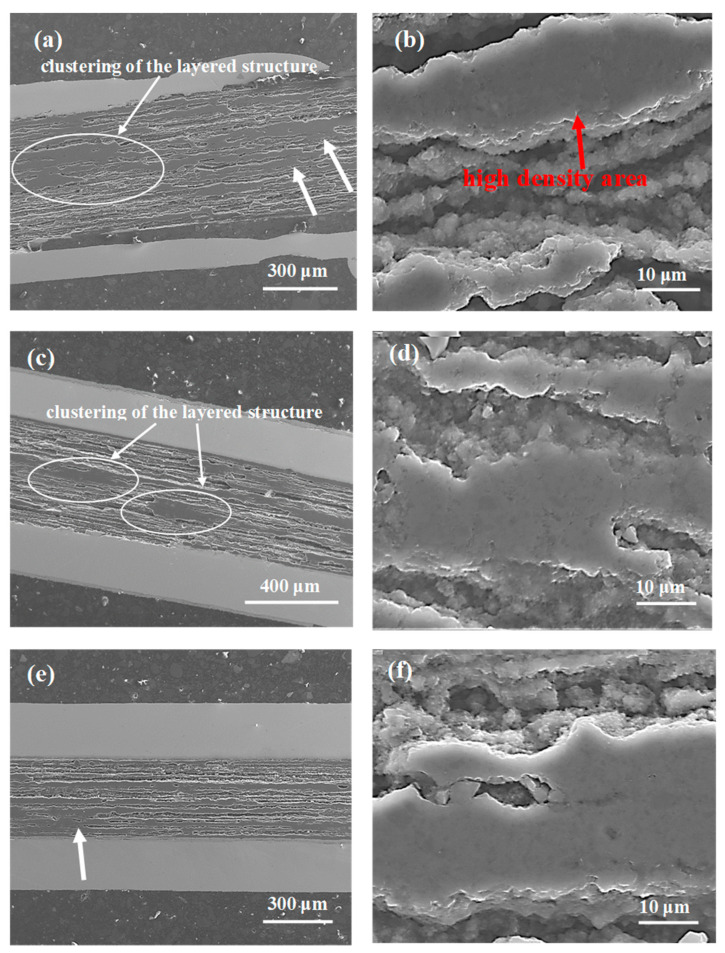
Longitudinal sections of MgB_2_ wires with boron grains of 1 µm for isostatic pressures of 0.1 MPa (**a**,**b**) sample A—annealing temperature of 680 °C; (**c**,**d**) sample B—annealing temperature of 700 °C; (**e**,**f**) sample C—annealing temperature of 740 °C. The white arrow indicates high-density areas that are not uniformly distributed in the wire structure.

The SEM images in Figure 1 show longitudinal sections of the samples from A to C, annealed in the temperature range from 680 °C to 740 °C under an isostatic pressure of 0.1 MPa. The longitudinal section allows for the best analysis of factors that significantly influence the engineering critical current density and *n*-value, e.g., connections between grains and homogeneity of the structure. The results indicate that the synthesis reaction in the sample from A to C is in the liquid state of Mg [9,10]. Figure 1a,c,e shows that the layered structure is clustered in all samples (white arrow), creating dense superconducting regions with a highly heterogeneous distribution. The homogeneous layered structure was shown to significantly improve the connections between grains, leading to an increase in critical current density [18]. However, the heterogeneous distribution of dense regions reduces the connection between grains and leads to a reduction in J_c_ and J_ce_. Based on the results of Kim et al. [25] and Motaman et al. [26], we can conclude that the inhomogeneous distribution of dense regions results in a reduction in the *n*-value. Figure 1b,d,f shows that the Mg liquid-state synthesis reaction creates high-density regions, significantly improving the connection between grains in regions with a highly dense structure. Unfortunately, these dense regions are short in length, as seen in Figure 1.

**Figure 2:**
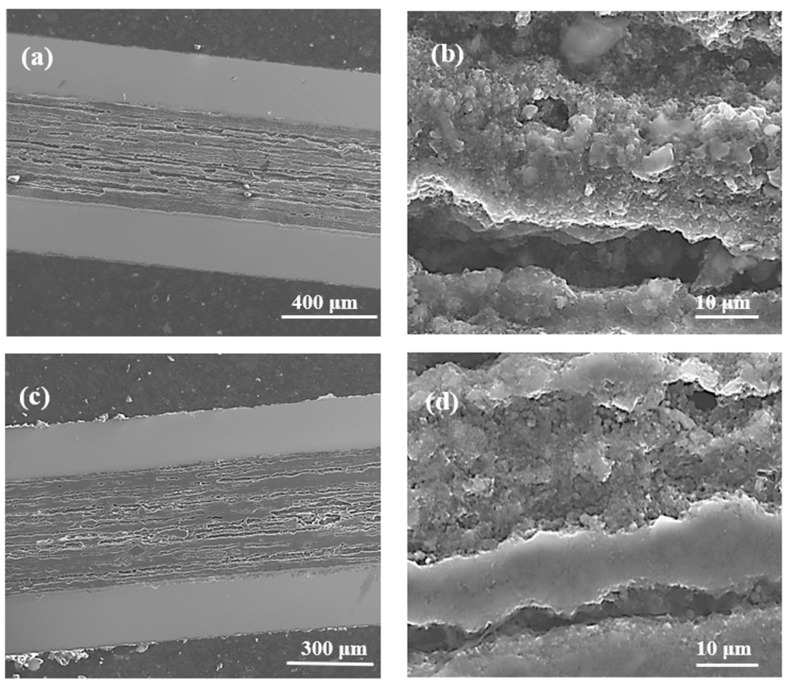
Longitudinal sections of MgB_2_ wires with boron grains of 1 µm at an isostatic pressure of 1.1 GPa (**a**,**b**) sample D—annealing temperature of 700 °C; (**c**,**d**) sample E—annealing temperature of 740 °C.

Thermal treatment of the samples under a high isostatic pressure of 1.1 GPa increases the melting point of magnesium up to 730 °C [9], indicating that the synthesis reaction in sample D takes place when Mg is in the solid state. Figure 2a shows that thermal treatment under a high isostatic pressure of 1.1 GPa and at an annealing temperature of 700 °C ensures a better homogeneity of the MgB_2_ material structure than sample B (700 °C and 0.1 MPa). Sample D has more layers with smaller thickness and fewer large dense regions, which leads to more connections between the grains and improves the *n*-value. Figure 2b shows that the layer structure in sample D (700 °C and 1.1 GPa) has a lower density than the layer structure of sample B (700 °C and 0.1 MPa). The relatively low layer density in sample D is due to the Mg solid-state synthesis reaction. Mg is in the liquid state in the thermal treatment of sample E (740 °C and 1.1 GPa) [9]. Figure 2c shows that sample E (740 °C and 1.1 GPa) has more homogeneously distributed dense layers than sample C (740 °C and 0.1 MPa), allowing for more connections between grains, increasing J_ec_, and improving the *n*-value.

Figure 2d indicates that the density of the layered structure in sample E is similar to the density of the layers in sample C (Figure 1f). The SEM images taken from sample D (700 °C and 1.1 GPa) and sample E (740 °C and 1.1 GPa) show that the Mg liquid synthesis reaction (Figure 2d) allows the formation of layers with a higher density than the Mg solid-state synthesis reaction (Figure 2b) under high isostatic pressure.

**Figure 3:**
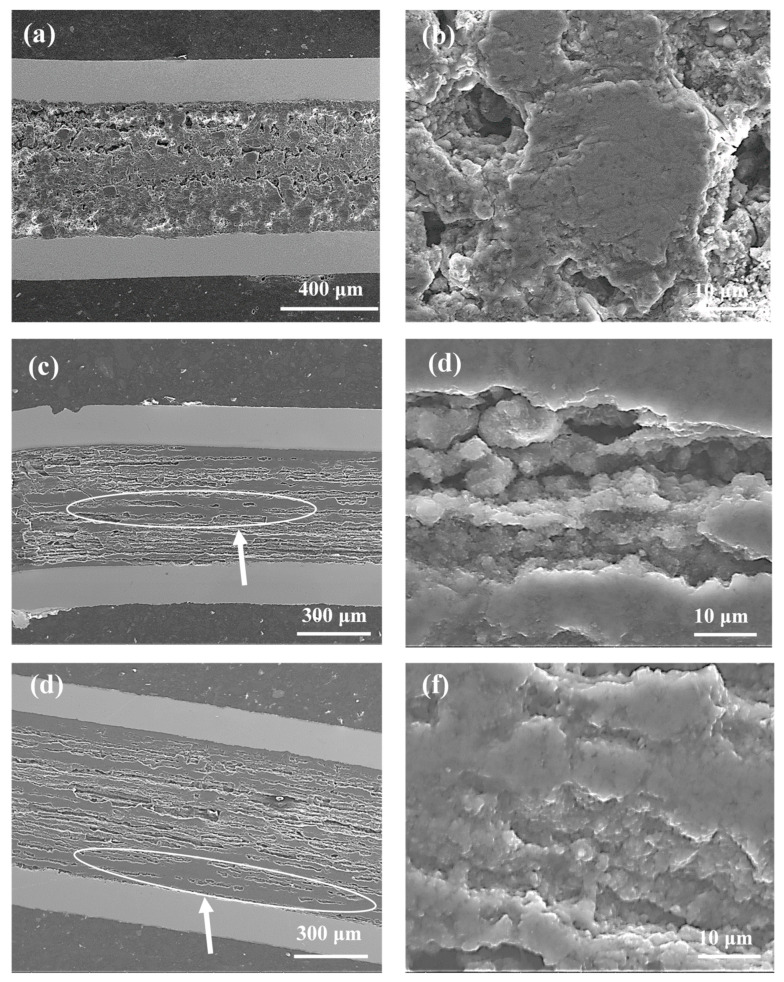
Longitudinal sections of MgB_2_ wires with boron grains of 1 µm and 0.25 µm for isostatic pressures of 0.1 MPa (**a**,**b**) sample F—annealing temperature of 680 °C; (**c**,**d**) sample G—annealing temperature of 700 °C; (**e**,**f**) sample H—annealing temperature of 740 °C. The white arrow indicates high-density areas that are not uniformly distributed in the wire structures.

The SEM images in Figure 3 represent the longitudinal sections for samples F to G (a mixture of boron powder with grain sizes of 1 µm and 0.25 µm). Figure 3a,b shows that the mixture of boron forms a grain structure, with islands up to a size of 150 µm, at an annealing temperature of 680 °C. The formation of island structures may result from the deployment of small boron grains. Small boron grains react faster than large boron grains at a low temperature, indicating that the large boron forms longitudinal connections, while the small boron forms transverse connections, decreasing the homogeneity of the superconducting material. The results in Figure 3c–f show that the annealing temperatures of 700 °C and 740 °C form a layered structure with inhomogeneous dense regions that reduce the number of connections between the grains. The results for samples A (boron—1 µm) and F (boron—1 µm and 0.25 µm) show that annealing at 680 °C creates different structures (sample A—layered structure and sample F—island structure), indicating that the MgB_2_ structure at low annealing temperature is dependent on particle size. The SEM images for samples B, C, G, and H show that annealing temperatures of 700 °C and 740 °C result in the formation of a similar structure, indicating that the particle size at a high annealing temperature does not affect the structure in terms of the number and distribution of dense regions.

**Figure 4:**
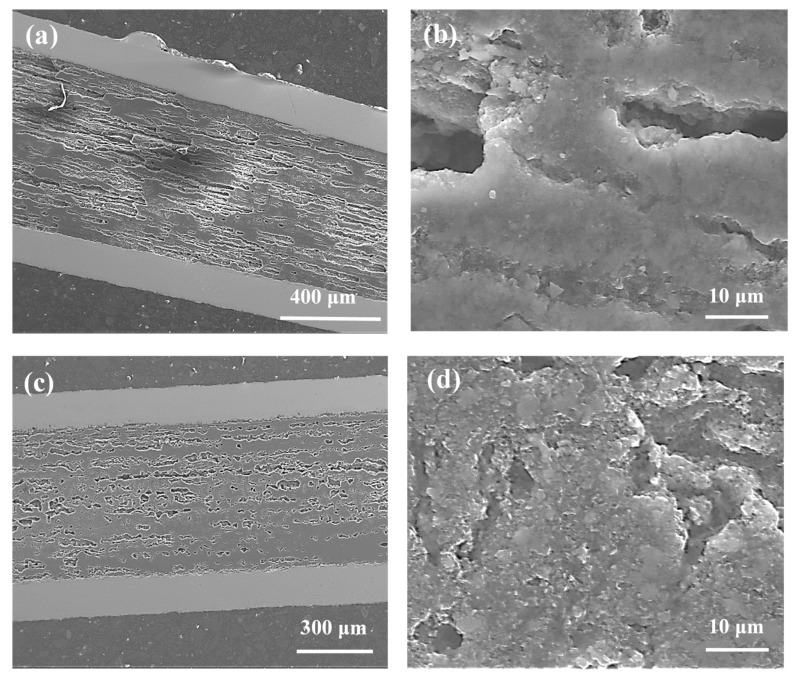
Longitudinal sections of MgB_2_ wires with boron grains of 1 µm and 0.25 µm for isostatic pressures of 1.1 GPa (**a**,**b**) sample I—annealing temperature of 700 °C; (**c**,**d**) sample J—annealing temperature of 740 °C.

The synthesis reaction for sample I was performed as Mg was in the solid state [9]. Figure 4a shows that the thermal treatment under an isostatic pressure of 1.1 GPa produces a greater number of dense regions with a better homogeneous distribution than sample G (700 °C and 0.1 MPa). Both samples have dense regions similar to those in Figure 3d and Figure 4b. When the results in Figure 2b are compared to those of Figure 4b, sample I (1 µm and 0.25 µm) can be seen to have a denser layered structure than sample D (1 µm), indicating that the small boron grains and the high isostatic pressure accelerate the synthesis reaction when Mg is in the solid state.

According to the results in [9], Mg is in the liquid state during the synthesis of sample J (740 °C and 1.1 GPa). The results in Figure 3d and Figure 4c show that sample J has a layered structure with better homogeneity and denser regions than sample H (740 °C and 0.1 MPa), allowing the formation of a greater number of connections between the grains. From a comparison of the results in Figure 4a with those in Figure 4c, sample J can be seen to have a layered structure with a better homogeneity and denser regions than sample I. However, sample I (Figure 4b) has higher density layers than sample J (Figure 4d). The SEM images in Figure 2c,d and Figure 4c,d show that high isostatic pressure and an annealing temperature of 740 °C contribute to the formation of a more homogeneous layered structure and a greater number of dense regions for the sample made of the boron mixture than the sample with boron grains 1 µm in size.

**Figure 5:**
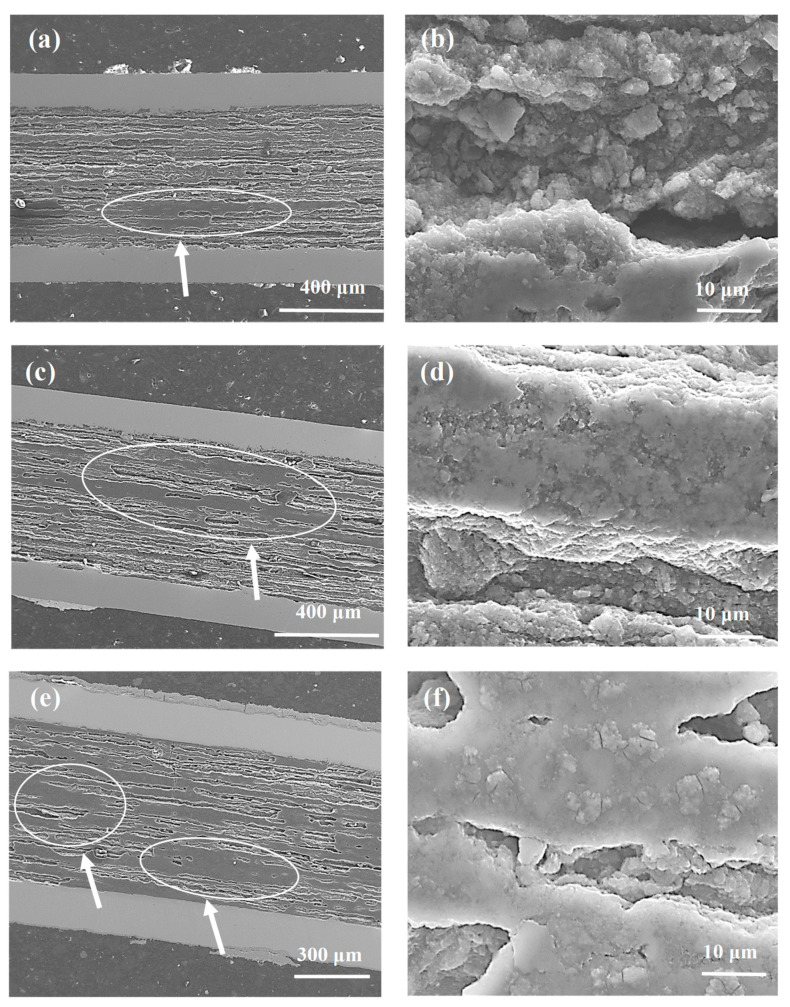
Longitudinal sections of MgB_2_ wires with boron grains of 0.25 µm at an isostatic pressure of 0.1 MPa (**a**,**b**) sample K—annealing temperature of 680 °C; (**c**,**d**) sample L—annealing temperature of 700 °C; (**e**,**f**) sample M—annealing temperature of 740 °C. The white arrow indicates high-density areas that are not uniformly distributed in the wire structures.

The micrographs in Figure 5 for samples with nano boron show that thermal treatment at temperatures ranging from 680 °C to 740 °C and under an isostatic pressure of 0.1 MPa create a heterogeneous distribution of the layered structure and dense regions (white arrows). This process reduces the number of connections between the grains. Figure 5b,d,f shows that increasing the annealing temperature from 700 °C to 740 °C for an isostatic pressure of 0.1 MPa leads to a significant increase in the density of the layered structure, indicating that annealing at a higher temperature enhances the connection between the grains.

**Figure 6:**
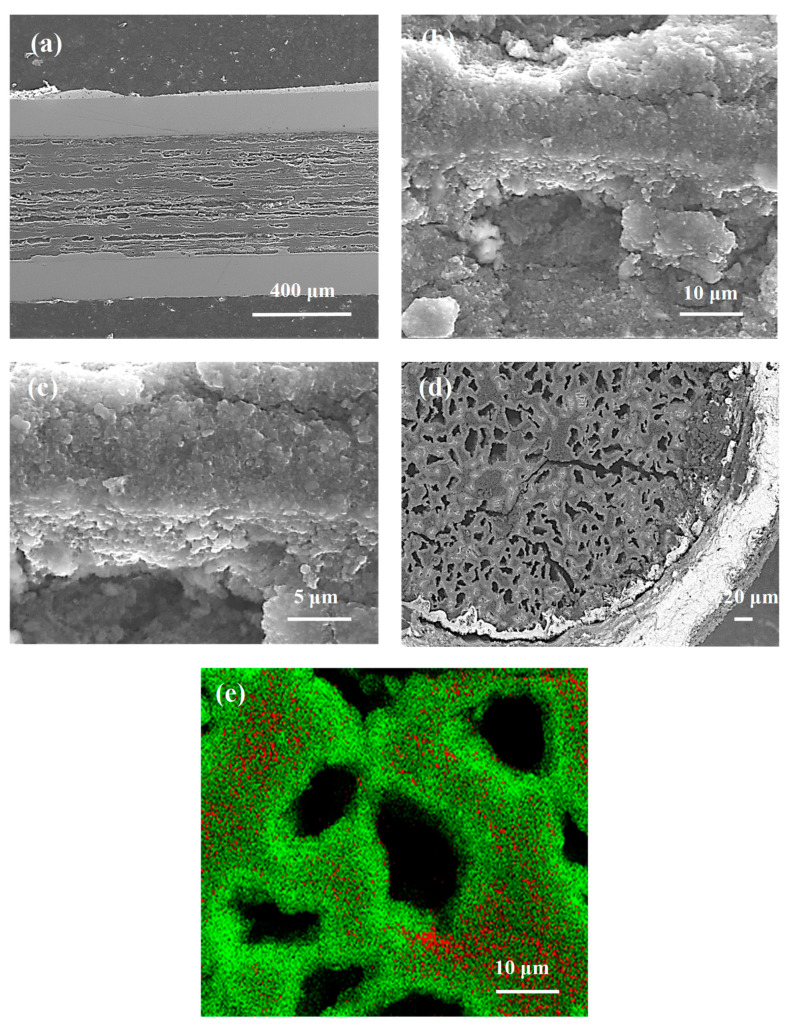
(**a**–**c**) The longitudinal section, (**d**) the cross-section—back scattering, and (**e**) the cross-section; EDX elemental maps for red—boron, green—magnesium for sample N (boron grains of 0.25 µm, isostatic pressure of 1.1 GPa, and annealing temperature of 700.

**Figure 7:**
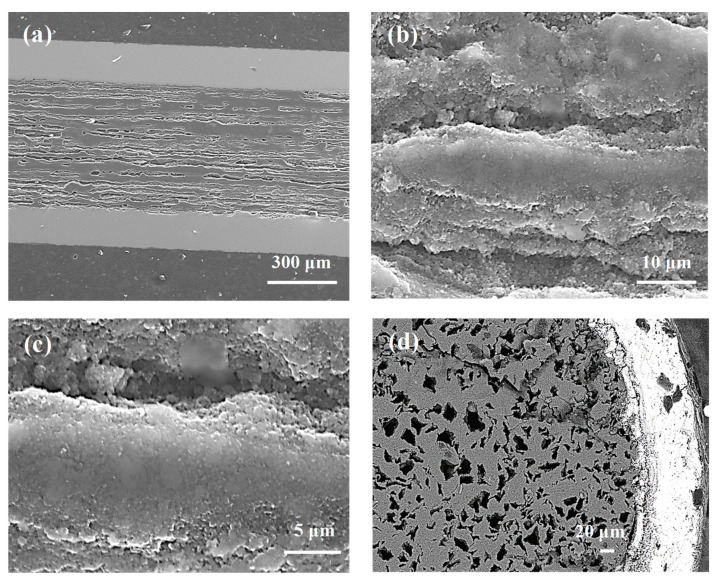
(**a**–**c**) The longitudinal section and (**d**) the cross section—backscattering analysis for sample O (boron grains of 0.25 µm for isostatic pressure of 1.1 GPa and annealing temperature of 740 °C).

The results in [9] show that the synthesis reaction in sample N is achieved for solid-state Mg. The results obtained from the longitudinal section (Figure 6a–c) and the cross section (Figure 6d—backscattering analysis and EDX elemental maps (Figure 6e) show that sample N has a large number of unreacted regions (e.g., B, Mg) and a small amount of superconducting material as thin layers around the voids, leading to a reduction in homogeneity for sample N and decreasing the number of connections between the grains and the critical parameters. According to the results in [9], Mg is in the liquid state during the thermal treatment of sample O under high isostatic pressure. Figure 7a–c (secondary electrons—SE) and Figure 7d (backscattering—(AsB)) show that a high annealing temperature and high isostatic pressure allow the formation of a large amount of superconducting material (no unreacted regions).

The SEM results from the longitudinal sections of the wires show that the core structures in Figure 1, Figure 3, and Figure 5 are very similar to each other, showing that the synthesis reaction in these samples is dependent mainly on the Mg state (solid/liquid) and less dependent on the density of unreacted material. The E, J, and O samples also look very similar, as seen in Figure 2c,d, Figure 4c,d, and Figure 7a–c. Thermal treatment under high pressure was carried out in the liquid Mg state for these samples. High isostatic pressure improves the homogeneity of the MgB_2_ structure, indicating that the synthesis reaction in these samples is dependent mainly on the liquid state of Mg and high isostatic pressure, and weakly dependent upon the density of unreacted material.

### 3.2. Analysis of Transport Measurements

#### 3.2.1. Thermal Treatment under Low Isostatic Pressure

**Figure 8:**
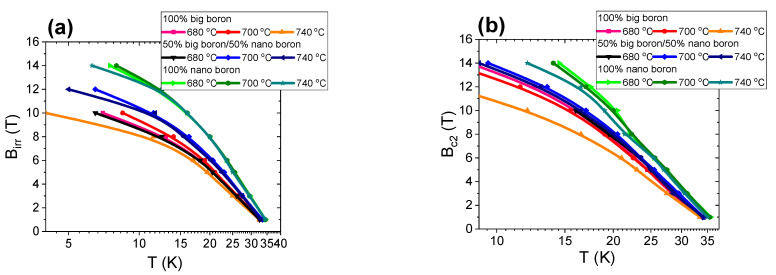
The temperature dependences of the irreversible magnetic field (B_irr_) (**a**) and the temperature dependences of the upper critical magnetic fields (B_c2_) for an isostatic pressure of 0.1 MPa (**b**).

Wang et al. [36] noted that the irreversible magnetic field is the maximum field in which MgB_2_ loses its ability to carry supercurrents. An irreversible magnetic field is also suggested to be able to increase by introducing effective pinning centers. Research shows that high-field pinning centers, such as dislocations, strains, and intragrain precipitations, effectively increase B_irr_ [37]. The results in Figure 8a for the A–C samples with large boron grains show that increasing the annealing temperature from 680 °C to 700 °C leads to an increase in B_irr_ by 10% at temperatures ranging from 4.2 K to 20 K. An additional 40 °C increase in the annealing temperature results in a reduction of B_irr_ by 20%. Figure 1 shows that the A–C samples have a similar layered structure and dense regions, indicating that increasing the annealing temperature is less effective in forming and accumulating high-field pinning centers for the sample with large boron grains. The transport measurements for the F–H samples (boron grains—1 µm and 0.25 µm) show that increasing the annealing temperature from 680 °C to 700 °C leads to an increase in B_irr_ of 20% (Figure 8a). A further increase in the annealing temperature by 40 °C does not increase B_irr_. The annealing temperature of 700 °C seems to allow the formation of more effective high-field pinning centers. Additionally, the research reveals that B_irr_ is strongly limited by the island structure (sample F—Figure 3b). The results for K–O samples show that increasing the annealing temperature from 680 °C to 740 °C does not change the B_irr_ value, suggesting that the formation and efficiency of high-field pinning are less dependent on the annealing temperature and might be more strongly dependent on nanoboron grains. The B_irr_ results indicate that nanosized boron grains increase B_irr_ more efficiently (by 30%) than large boron grains. Nanoboron forms much better high-field pinning centers than large boron for the liquid state of Mg during the synthesis reaction (despite the lower filling density of the unreacted material).

Kazakov et al. [38] show that a decrease in superconducting coherence length leads to an increase in B_c2_. The superconducting coherence length decreases with structural defects, e.g., substitution of C for B in the MgB_2_ material [38,39]. The transport measurement results in Figure 8b show that samples with a large boron have a similar B_c2_. Only sample E (large grains) after heat treatment at 740 °C has a significantly lower B_c2_ by 20%. These results indicate that increasing the annealing temperature in samples with large boron grains slightly increases the density of structural defects and slightly reduces the coherence length. The results in Figure 8b for the sample with nanoboron show that increasing the annealing temperature from 700 °C to 740 °C does not change B_c2_, indicating that the annealing temperature slightly affects the formation of structural defects and the coherence length. When the transport measurement results for the samples with large boron and nanoboron are compared, nanoboron can be seen to significantly increase B_c2_ by 25% (Figure 8b), revealing that nanoboron creates more structural defects than large boron. The results in Figure 8a,b and the results in [15] show that the B_irr_ and B_c2_ values of our wires are similar to the B_irr_ and B_c2_ values obtained for undoped MgB_2_ wires with nanoboron grains, suggesting that our MgB_2_ wires have high critical parameters.

**Figure 9:**
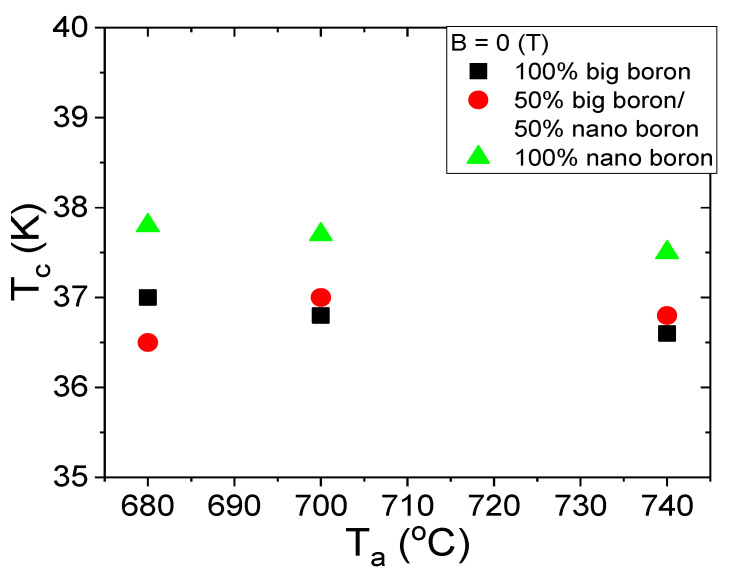
The annealing temperature (T_a_) dependences of the critical temperature (T_c_) for an isostatic pressure of 0.1 MPa and B = 0 T.

The T_c_ is dependent on several factors, e.g., lattice constants, annealing temperature, starting material, admixtures, and material purity [38,39]. Moreover, Buzea et al. reported that shrinkage of MgB_2_ material reduces the T_c_ by 1 K [40]. The results in Figure 9 show that increasing the annealing temperature from 680 °C to 740 °C slightly affects the T_c_ value. In addition, the results in Figure 9 show that the samples made with only nanoboron have a T_c_ of 0.8 K higher than the samples made with large boron and boron mixtures (1 µm and 0.25 µm). These results show that the grain size of boron has a more significant influence on T_c_ than the annealing temperature. When comparing our results in Figure 9 with the results in [15], we see that the T_c_ of our MgB_2_ wires with nanoboron grains is approximately 1.5 K higher than the T_c_ of undoped MgB_2_ wires with nanoboron grains [15].

**Figure 10:**
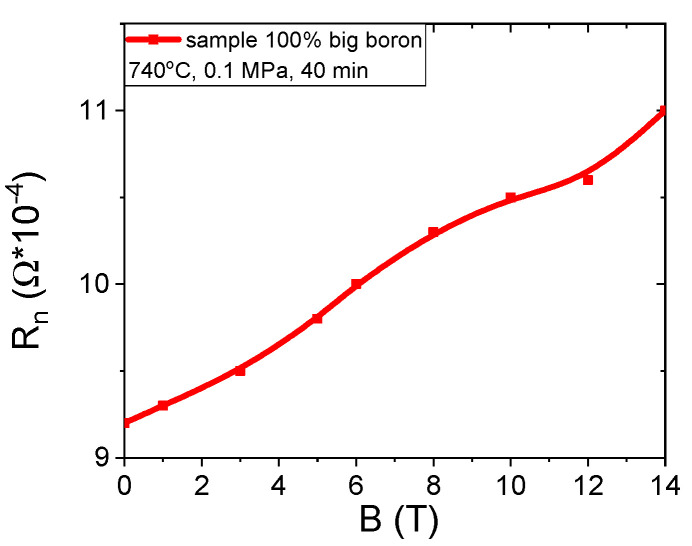
The magnetic field (B) dependences of the normal state resistance for sample C.

The results in Figure 10a show that an annealing temperature of 740 °C results in a 15% increase in normal state resistance (R_n_) when the magnetic field is increased from 0 T to 14 T. In the A and B samples heated at 680 °C and 700 °C, no increase was observed in normal state resistance as the magnetic field increased because sample C was annealed at a higher temperature, indicating that magnetoresistance occurs due to the Mg and B compounds containing Fe and does not depend on unreacted Mg. Magnetoresistance caused by unreacted Mg was not seen even in samples A and B that were annealed at a lower temperature. The transport measurements in the zero magnetic field show that the K–L samples have lower resistance (30% less) in the normal state than the A–C and F–H samples, indicating that nanoboron creates a larger number of connections between the grains than large boron and a mixture of boron.

**Figure 11:**
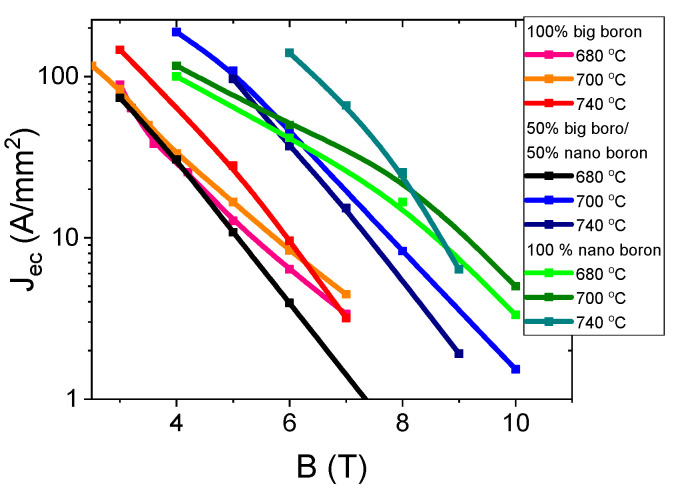
Transport J_ec_-B curves for undoped MgB_2_ wires in a perpendicular magnetic field at an isostatic pressure of 0.1 MPa at 4.2 K.

The results in Figure 11 for samples A–C (grain size of 1 µm) show that increasing the annealing temperature from 680 °C to 740 °C significantly increases the J_ec_ by approximately 50% in the magnetic field range from 3 T to 6 T. The high annealing temperature in MgB_2_ wires with large boron grains seems to form mainly on surface pinning centers [29]. J_ec_ was determined with the ratio J_ec_ = I_c_/S (S—wire cross-section) [29]. The transport results for sample F–H (mixture of boron grains—1 µm and 0.25 µm) show that increasing the annealing temperature from 680 °C to 700 °C increases J_ec_ by one order of magnitude (Figure 11). A further increase in annealing temperature leads to a slight reduction in J_ec_. An annealing temperature of 700 °C for samples with a mixture of boron grains significantly increases the density of all types of pinning centers [29,37]. The results for the K–M samples (nanoboron—0.25 µm) show that increasing the annealing temperature from 680 °C to 740 °C significantly increases J_ec_ by 60% in the magnetic field range from 6 T to 8 T and significantly reduces J_ec_ by 40% in magnetic fields above 8 T (Figure 11). These results indicate that the high annealing temperature in MgB_2_ wires with nanoboron mainly forms surface pinning centers. However, a lower annealing temperature in the sample with nanoboron more effectively creates high-field pinning centers. Based on the results in Figure 11, the mixture of boron grains (1 µm and 0.25 µm) can be considered to significantly increase the J_ec_ at all annealing temperatures in comparison to the sample with large boron grains (1 µm) because the addition of nanoboron increases the density of all types of pinning center [29,37]. A comparison of the results for samples with the boron mixture with the results for samples with nanoboron reveals that only nanoboron allows for high J_ec_ in medium and high magnetic fields, and it meets the applicability requirements (e.g., 100 A/mm^2^, 50 A/mm^2^, and 20 A/mm^2^). Currently, studies show that the highest J_ec_ is obtained in MgB_2_ wires made by internal Mg diffusion (IMD) [41,42]. Our sample M (nanoboron—740 °C and 0.1 MPa) has J_ec_ 100 A/mm^2^ at 7 T. This J_ec_ is similar to the J_ec_ of doped MgB_2_ wire (PIT) [43] and higher than the J_ec_ of undoped MgB_2_ IMD wire (IMD) [44].

**Figure 12:**
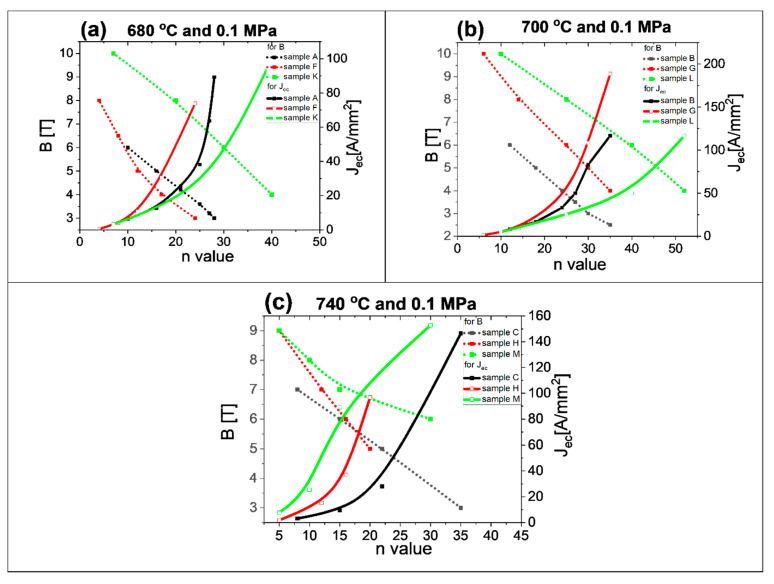
The dependence of the *n*-value on the magnetic field (B) perpendicular to the wire axis in undoped MgB_2_ wires for an isostatic pressure of 0.1 MPa: (**a**) annealing temperature of 680 °C; (**b**) annealing temperature of 700 °C; (**c**) annealing temperature of 740 °C.

The *n*-value determines J_ec_, which can be used in superconducting coils [27]. The *n*-value was calculated using the formula in [45]. Our results in Figure 12 represent the relationships between the *n*-value, magnetic field, and engineering critical current density. This analysis helps identify which MgB_2_ wire can be used to build low-, middle- and high-field superconducting coils. The results in Figure 12a for an annealing temperature of 680 °C and isostatic pressure of 0.1 MPa indicate that nanoboron grains allow the highest *n*-value, depending on B and J_ec_. The SEM images in Figure 5a show that the sample with nanoboron grains has a more homogeneous structure (e.g., layers have a lower thickness and fewer clustered layers) than a sample made with large boron grains (Figure 1a), leading to an increase in the number of connections between grains and a high *n*-value and J_ec_. The research shows that the lowest *n*-value belongs to the sample made with the mixture of boron grains (1 µm and 0.25 µm), indicating that the island structure most reduces the *n*-value and J_ec_. Figure 3a shows that sample F has the least homogeneous structure. Parizh et al. [27] indicated that an *n*-value of 20 allows the use of only 50% of the J_ec_ value. The results for the annealing temperature of 680 °C indicate that MgB_2_ wires with large boron grains can be used for the construction of low-field superconducting coils (for J_ec_—40 A/mm^2^). However, MgB_2_ wires with nanoborons can be used to build medium-field superconducting coils.

The transport results after annealing at 700 °C and isostatic pressure of 0.1 MPa (Figure 12b) show that the MgB_2_ wire with nanoboron grains has a higher *n*-value than the wires with large boron grains and a mixture of boron grains, e.g., *n*-value of 40 in 6 T for nanoboron, *n*-value of 12 in 6 T for large boron. The research also shows that the addition of nanoboron grains (sample G) also increases the *n*-value (Figure 12b). The SEM images in Figure 1c, Figure 3c, and Figure 5c show that the sample with nanoboron grains has a structure with thinner layers and fewer clustered layers than the sample with large and mixed boron grains, also leading to an improvement in *n*-value. The results in Figure 12b show that an annealing temperature of 700 °C allows fabrication of only low-field superconducting coils from MgB_2_ wires with large boron grains. However, research shows that MgB_2_ wires with nanoboron grains, after annealing at 700 °C, can be used to build high-field superconducting coils (up to 7.5 T).

The transport measurements in Figure 12c show that the annealing temperature of 740 °C and isostatic pressure significantly increase the *n*-value in nanoboron grain MgB_2_ wires only under low and medium magnetic fields and slightly increase the *n*-value under high magnetic fields compared to the MgB_2_ wire produced from mixtures of boron grains.

A higher annealing temperature improves the homogeneity of the structure in MgB_2_ wires for large boron and a mixture of boron (Figure 1e and Figure 3e), leading to an improvement in the n-value. However, the annealing temperature of 740 °C reduces the homogeneity of MgB_2_ wire with nanoboron (Figure 5e). The results of Figure 12c show that MgB_2_ wires with large boron and a mixture of boron can be used to build medium field superconducting coils (up to 5 T).

The increase in the annealing temperature from 680 °C to 740 °C for MgB_2_ wires with large boron and a mixture of boron leads to an increase in the *n*-value. Additionally, the measurements show that increasing the annealing temperature from 680 °C to 740 °C for samples with nanoboron grains increases the *n*-value in low and medium magnetic fields and reduces the *n*-value under high magnetic fields, indicating that the *n*-value is related to J_ec_ and pinning centers because sample M after heat treatment at 740 °C has the highest J_ec_ in low and medium magnetic fields and a significantly lower J_ec_ in high magnetic fields than sample L (700 °C). We obtained an *n*-value of 30 at 7.5 T for the MgB_2_ wire with nanoboron grains (700 °C and 0.1 MPa). Li et al. [43] found that the *n*-value was 30 under 6 T for undoped MgB_2_ wires made by conventional continuous tube forming/filling (CTFF). Moreover, research in [25] showed that undoped MgB_2_ wires made by the IMD technique had an *n*-value of 30 under 8 T, indicating that our undoped MgB_2_ wires have J_ec_ and *n*-values similar to J_ec_ and *n*-values of the best undoped MgB_2_ wires.

Our research shows that nanoboron after a reaction with Mg in the liquid state has a great influence on critical parameters (B_irr_, B_c2_, T_c_, J_ec_, and *n*-value). In addition, we proved that the homogeneous structure of MgB_2_ material, e.g., uniform distribution of voids, thickness layers, and material density of MgB_2_, significantly affected the J_ec_ and *n* value in all samples (large boron, boron mixture, and small boron).

#### 3.2.2. Thermal Treatment at 700 °C under High Isostatic Pressure

**Figure 13:**
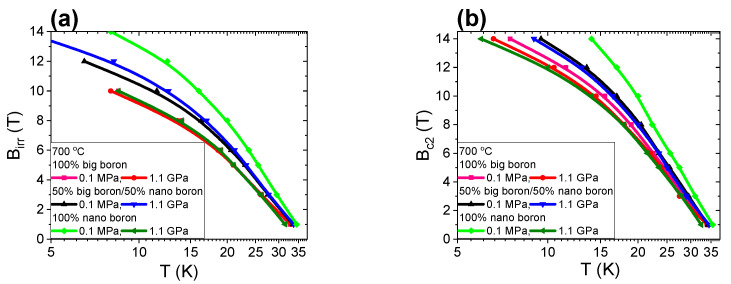
Temperature dependence of the irreversible magnetic field (B_irr_) (**a**) and of the upper magnetic field (B_c2_) for an annealing temperature of 700 °C (**b**).

The results presented in [9] show that the melting point of Mg is approximately 730 °C for an isostatic pressure of 1.1 GPa. Thus, the synthesis reactions in samples D, I, and N are in the solid state of Mg. However, Mg was in the liquid state for the samples B, G and L thermally treated at a low isostatic pressure of 0.1 MPa. The transport results for large boron grains (1 µm) show that thermal treatment under high isostatic pressure does not improve B_irr_ (Figure 13a), indicating that the 1.1 GPa isostatic pressure does not improve or increase the density of high-field pinning centers in MgB_2_ wires with large boron grains for Mg solid-state synthesis reactions.

Further studies show that high isostatic pressure annealing for sample I with the boron mixture (1 µm and 0.25 µm) slightly increases B_irr_ (Figure 13a), indicating that the isostatic pressure of 1.1 GPa weakly improves the density of high-field pinning centers in MgB_2_ wires with the boron mixture when Mg is in the solid state. Transport measurements for sample N with nanoboron grains (0.25 µm) show that thermal treatment under high isostatic pressure significantly reduces B_irr_ by 30% (Figure 13a). The SEM results in Figure 6 show that the significant reduction in B_irr_ is associated with a small superconducting phase and the large amount of unreacted B and Mg in sample N. These results show that thermal treatment under high isostatic pressure for the solid state of Mg weakly forms high-field pinning centers in MgB_2_ wires with a lower unreacted material density. The research shows that the mixture of boron grains and HIP process increases B_irr_ by 15% compared to large boron grains for the Mg solid-state synthesis reaction and the same density of unreacted material (1.5 g/cm^3^). Studies show that nanoboron grains and the HIP process do not increase B_irr_ in comparison to large boron grains for the Mg solid-state synthesis reaction and the 8% lower filling density of unreacted material (1.38 g/cm^3^). These results indicate that the synthesis reaction of MgB_2_ material in the solid state of Mg is more dependent on the density of unreacted material than the particle size of the boron. The transport measurements in Figure 13b show that heat treatment under high isostatic pressure slightly increases B_c2_ in samples with large boron grains and a mixture of boron grains, indicating that the isostatic pressure of 1.1 GPa weakly increases the density of the structural defects, which leads to a reduction in the coherence length. Studies for nanoboron grains (sample N) show that high isostatic pressure annealing leads to a significant reduction in B_c2_ by 30% (Figure 13b). This significant reduction in Bc2 is the result large amount of unreacted B and Mg, revealing that Bc2 is dependent on the density of the unreacted material for the Mg solid-state synthesis reaction and less dependent on boron grain size. However, the B_c2_ value for the Mg liquid-state synthesis reaction is dependent mainly on boron grain size and less dependent on the density of the unreacted material.

**Figure 14:**
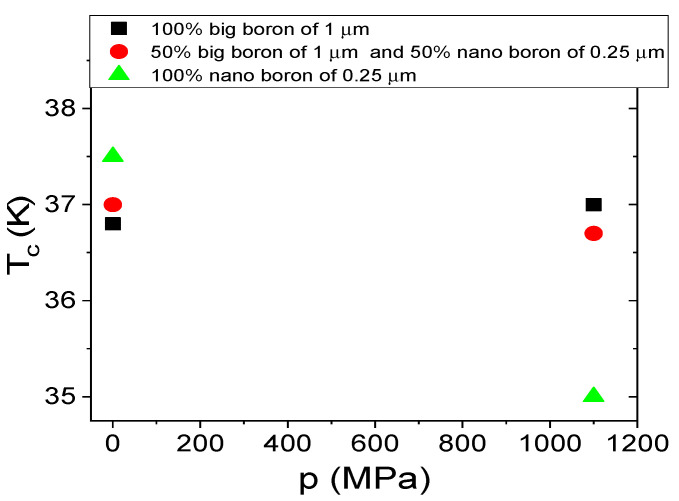
The isostatic pressure (p) dependences of the critical temperature (T_c_) for an annealing temperature of 700 °C.

The transport measurements in Figure 14 show that the thermal treatment under high pressure does not affect the critical temperature for MgB_2_ wires with large boron and mixtures of boron after the HIP process. However, the sample with nanoboron with a lower filling density of the unreacted material has a lower T_c_ by approximately 2.5 K for the solid-state synthesis reaction of Mg and annealing at high isostatic pressure. This result is due to the - lower amount of superconducting phase, unreacted B and Mg, or the nonstoichiometric superconducting phase.

**Figure 15:**
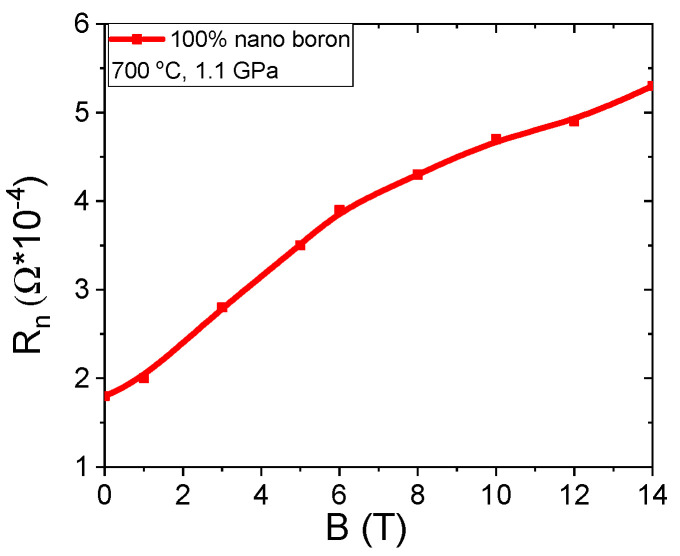
The effect of the magnetic field (B) on the normal state resistance for sample N.

The results in Figure 15 show that increasing the magnetic field from 0 T to 14 T leads to a threefold increase in the normal state resistance. Such a large increase in magnetoresistance is caused by the large amount of unreacted Mg and B, indicating that transport measurements allow for the detection of unreacted particles, e.g., Mg. The transport measurements for samples with large boron grains show that thermal treatment at high isostatic pressure reduces the normal state resistance by approximately 40%. Further studies show that annealing under high isostatic pressure leads to a reduction in the normal state resistance by 50% in MgB_2_ wires with the boron grain mixture. Transport measurements for nanoboron grains show that thermal treatment under an isostatic pressure of 1.1 GPa reduces R_n_ by 70%. However, such a large decrease in R_n_ is not only the result of thermal treatment under high isostatic pressure but also of the remnant of unreacted pure Mg, which has a very low resistivity. The reduction of R_n_ is very advantageous because it indicates that the HIP process increases the number of connections between grains.

**Figure 16:**
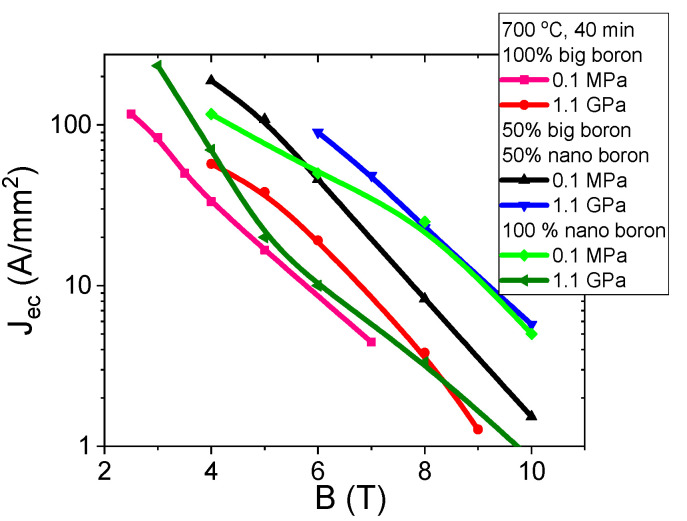
Transport J_ec_-B curves at 4.2 K for undoped MgB_2_ wires in perpendicular magnetic field for annealing temperature of 700 °C.

The results in Figure 16 show that thermal treatment under high isostatic pressure increases J_ec_ by 50% in MgB_2_ wires with large boron grains. Transport measurements for MgB_2_ wires with a boron mixture of grains and thermal treatment under an isostatic pressure of 1.1 GPa increases J_ec_ by 65% (Figure 16). The SEM photographs (Figure 1c,d and Figure 2a,b, Figure 3c,d, Figure 4a,b) and the results of B_irr_ (Figure 13a) indicate that J_ec_ is improved with better homogeneity in the structure after the HIP process, e.g., more connections between grains and thinner layered structures. Figure 16 shows that heat treatment under high isostatic pressure significantly decreases J_ec_ by one order of magnitude in MgB_2_ under medium and high magnetic fields for wires with nanoboron grains, due to the large amount of unreacted Mg and B material, lower structural homogeneity, and lower filling density of unreacted materials. Studies show that samples with a higher density of unreacted material (8%) have a higher J_ec_ during the synthesis reaction under high isostatic pressure. However, a sample with unreacted material with a lower filling density after the synthesis reaction under high isostatic pressure has a significantly lower J_ec_, despite being made of nanoboron grains, indicating that J_ec_ is dependent on the density of the unreacted material and independent of boron grain size during the solid-state Mg synthesis reaction.

**Figure 17:**
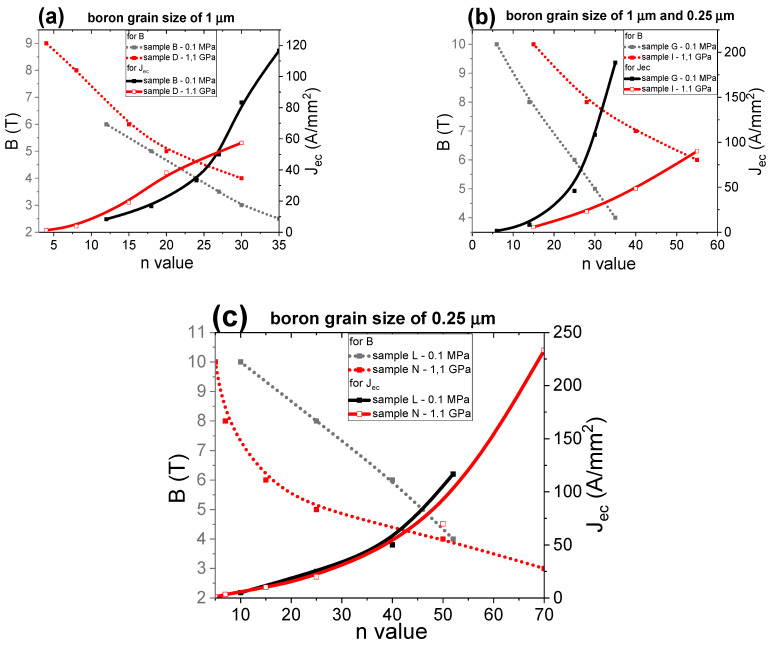
The dependence of the *n*-value on the magnetic field (B) perpendicular to the wire axis in undoped MgB_2_ wires for an annealing temperature of 700 °C: (**a**) boron grain size of 1 μm, (**b**) boron grain mixture of 1 μm and 0.25 μm, and (**c**) boron grain size of 0.25 μm.

Figure 17 shows the dependence of the *n*-value on the magnetic field (B) and J_ec_. These results show that the HIP process and Mg in the solid state weakly increase the *n*-value for MgB_2_ wires with large boron grains (Figure 17a). The results in Figure 17b show that the heat treatment under high isostatic pressure and the Mg solid-state synthesis reaction significantly increase the *n*-value for MgB_2_ wires with a boron grain mixture, indicating that the nanoboron grains, due to their higher density of unreacted material, and high isostatic pressure lead to an improvement in the *n*-value. The results for the sample with nanoboron grains (Figure 17c) indicate that annealing under an isostatic pressure of 1.1 GPa leads to a significant reduction in the *n*-value under medium and high magnetic fields. Such a significant decrease in the *n*-value is a result of the lower filling density of unreacted material in sample N for the Mg solid-state reaction. The results in Figure 17 indicate that the *n*-value is dependent mainly on the density of unreacted material and less dependent on the grain size for the solid-state Mg synthesis reaction. The research presented by Wan et al. [46] shows that 2% carbon-doped MgB_2_ PIT wires with nanoboron grains and an Nb barrier after pressure treatment have an *n*-value of 50 at 7 T. We obtain an *n*-value of 50 at 6.5 T for undoped MgB_2_ wires with boron mixture grains (1 µm and 0.25 nm) under high isostatic pressure annealing, indicating that our undoped MgB_2_ wires have high *n*-values and J_ec_. Our research showed that the density of unreacted material during the Mg solid-state synthesis reaction significantly influences the critical parameters (T_c_, B_irr_, B_c2_, J_ec_, and *n*-value). Moreover, we observed that the HIP process improved the homogeneity of the MgB_2_ material structure in all samples (large boron and boron mixture), which led to a significant improvement in the J_ec_ and *n* values.

#### 3.2.3. Thermal Treatment at 740 °C under High Isostatic Pressure

**Figure 18:**
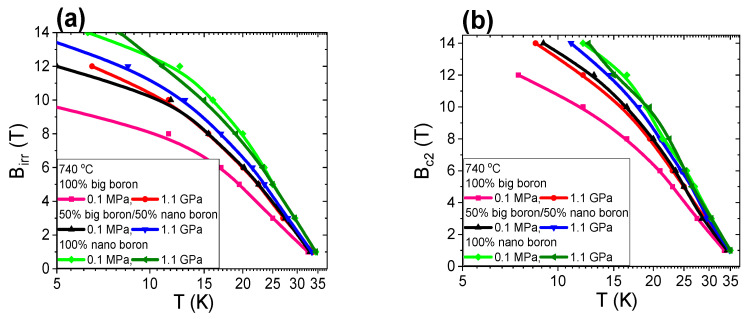
Temperature dependence of the irreversible magnetic field (B_irr_) (**a**) and the temperature dependences of the upper magnetic fields (B_c2_) (**b**) for an annealing temperature of 740 °C.

The results in [9] show that Mg is in the liquid state for thermal treatment under an isostatic pressure of 1.1 GPa and an annealing temperature of 740 °C. The results in Figure 18a show that high isostatic pressure annealing increases B_irr_ by 30% for MgB_2_ wires with large boron grains. Furthermore, transport measurements also showed that thermal treatment under high isostatic pressure increases B_irr_ by 10% in MgB_2_ wires with mixtures of boron grains (Figure 18a). The following measurements show that the HIP process does not increase B_irr_ in the MgB_2_ wires with nanoboron grains (Figure 18a), indicating that high isostatic pressure more effectively increases the density of high-field pinning centers in MgB_2_ wires with large boron grains during the synthesis reaction in which Mg is in the liquid state. The transport measurements show that the HIP process slightly increases the density of high-field pinning centers for MgB_2_ wires with nanoboron grains during the synthesis reaction in which Mg is in the liquid state. The results of sample N (700 °C and 1.1 GPa—Figure 13a) and the results of sample O (740 °C and 1.1 GPa—Figure 18a) show that a higher B_irr_ for a less unreacted material allows us to obtain a synthesis reaction for Mg in the liquid state, indicating that high-field pinning centers for unreacted material with a lower filling density are efficiently formed during the HIP process when only Mg is in the liquid state. The measurement results in Figure 13a and Figure 18a for the wires with nanoboron show that increasing the annealing temperature from 700 °C to 740 °C for 0.1 MPa isostatic pressure does not increase B_irr,_ indicating that increasing the annealing temperature from 700 °C to 740 °C and 0.1 MPa isostatic pressure slightly improves the high-field centers in MgB_2_ wires with nanoboron grains.

The results in Figure 18b show that high-pressure heat treatment at 740 °C increases B_c2_ by 20% for MgB_2_ wires with large boron grains. In Figure 18b, we see that a high isostatic pressure slightly increases B_c2_ for MgB_2_ wires with nanoboron grains, indicating that Mg in the liquid state and the HIP process create a greater number of structural defects, which leads to a greater reduction in the coherence length in MgB_2_ wires with large boron grains than in MgB_2_ wires with nanoboron grains. Transport measurements show that the highest B_c2_ is obtained for MgB_2_ wires with nanoboron grains compared to MgB_2_ wires with large boron grains, indicating that the nanoboron grains create the largest number of structural defects during the synthesis reaction when Mg is in the liquid Mg state. Unreacted material density has little effect on B_c2_ during the synthesis reaction of Mg in the liquid state.

**Figure 19:**
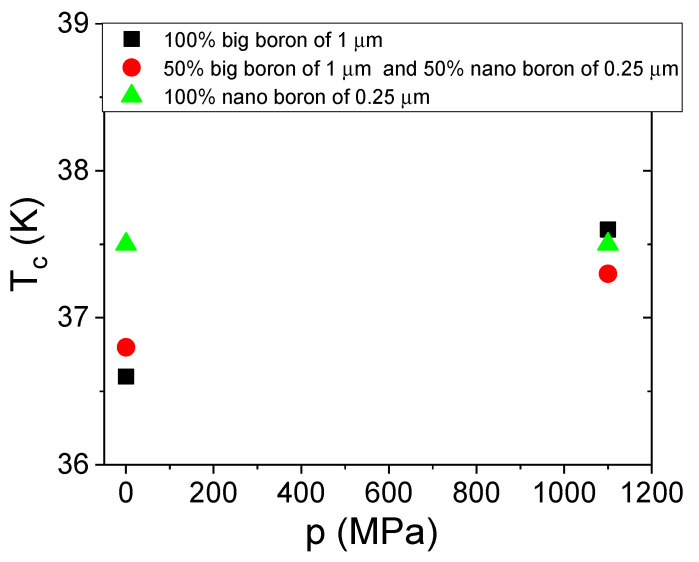
The isostatic pressure (p) dependences of the critical temperature (T_c_) for an annealing temperature of 740 °C.

The transport results in Figure 19 show that the HIP process and the annealing temperature of 740 °C allow for an increase in the critical temperature (T_c_) in MgB_2_ wires with large boron grains and a mixture of boron grains. These results indicate that thermal treatment of Mg in the liquid state under high isostatic pressure is required for a higher T_c_ in MgB_2_ wires with large boron grains, even for a higher density of unreacted material. A higher T_c_ indicates that a high annealing temperature (740 °C—Figure 19) allows us to obtain a better superconducting phase for wires with large boron grains than a lower thermal treatment temperature under an isostatic pressure of 1.1 GPa (700 °C—Figure 14). The results for samples with nanoboron grains also indicate that thermal treatment at 740 °C under an isostatic pressure of 1.1 GPa does not improve the T_c_ (Figure 19). The results in Figure 14 and Figure 19 for MgB_2_ wires with nanoboron grains show that the lower filling density of unreacted material requires a higher annealing temperature to obtain a high T_c_ during the HIP process.

**Figure 20:**
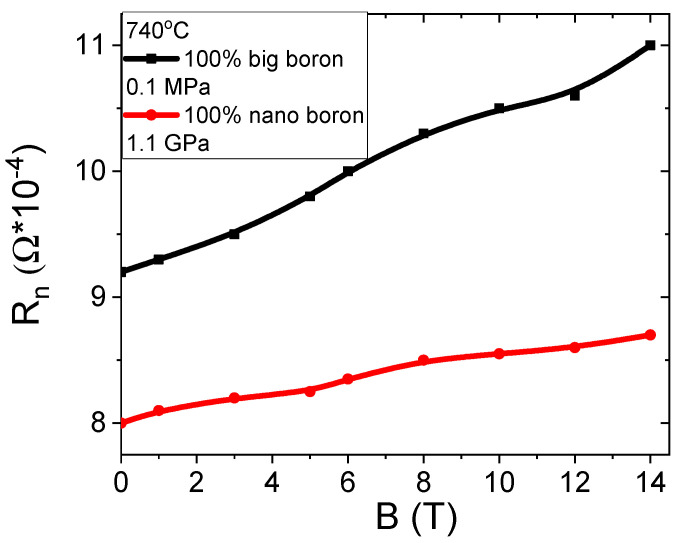
The magnetic field (B) dependences of normal state resistance for samples C and N.

In the previous section, we showed that the magnetoresistance in sample C is formed by intermetallic phases, e.g., Mg, B, and Fe (Figure 20). The results in Figure 20 for sample O (nanoboron grain) show that increasing the magnetic field from 0 T to 14 T leads to an increase in the normal state resistance in the range from 10% to 15%, indicating that sample O has a small amount of unreacted Mg and B. The SEM analysis using the AsB detector (Figure 7d) did not display the presence of any unreacted Mg in sample O. When the results of the magnetoresistance of samples N (66%) with O (from 10% to 15%) in Figure 15 and Figure 20 are compared, we can conclude that the unreacted material with a lower filling density requires thermal treatment at a higher temperature under an isostatic pressure of 1.1 GPa, leading to a significant reduction in the magnetoresistance in MgB_2_ wires with nanoboron grains. The transport measurements for the annealing temperature of 740 °C and increasing isostatic pressure from 0.1 MPa to 1.1 GPa showed that normal state resistance increases (for B = 0 T) by 12% for MgB_2_ wires with large boron grains and by 6% for MgB_2_ wires with nanoboron grains, indicating that thermal treatment in the liquid state of Mg leads to a reduction in the connections between the grains. Measurements of MgB_2_ wires with the mixture of boron grains show that the high isostatic pressure and the annealing temperature of 740 °C reduce R_n_ by 9%, indicating that the mixture of boron grains allows for more intergranular connections. Figure 4c shows that sample J has the highest number of connections between grains among all samples. However, the research (740 °C and 1.1 GPa) shows that the sample with nanoboron grains has the lowest R_n_ value by approximately 25% compared to the R_n_ for the samples with large grains and a mixture of boron grains, showing that nanoboron grains allow for the highest number of connections between grains, even for the lower filling density of unreacted material during the synthesis reaction in the liquid Mg state.

**Figure 21:**
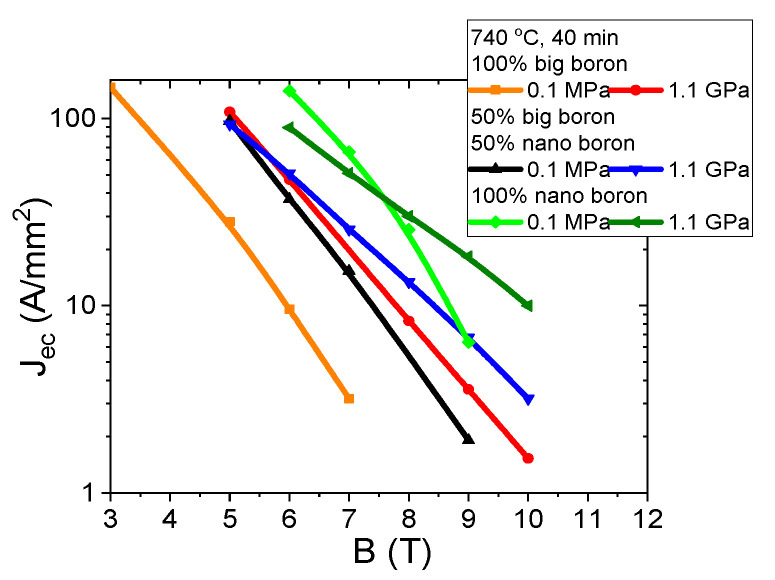
Transport J_ec_-B curves at 4.2 K for undoped MgB_2_ wires in perpendicular magnetic fields for an annealing temperature of 740 °C.

The results in Figure 21 show that an annealing temperature of 740 °C and an isostatic pressure of 1.1 GPa increases J_ec_ by one order of magnitude in MgB_2_ wires with large boron grains. Transport measurements show that thermal treatment at 740 °C under an isostatic pressure of 1.1 GPa improves J_ce_ under medium and high magnetic fields in MgB_2_ wires containing mixtures of boron grains (Figure 21). Additional measurements show that a high annealing temperature and high isostatic pressure increase J_ec_ by 70% under high magnetic fields in MgB_2_ wires with nanoboron grains. Our measurements also show that an annealing temperature of 740 °C and an isostatic pressure of 1.1 GPa lead to a reduction in J_ec_ by 35% under medium magnetic fields in MgB_2_ wires with nanoboron grains. The results in Figure 21 indicate that high isostatic pressure most effectively increases the density of high-field pinning centers in MgB_2_ wires with nanoboron grains for the synthesis reaction for Mg in the liquid state. Moreover, the results in Figure 21 indicate that an annealing temperature of 740 °C and a low isostatic pressure most effectively increase the density of medium field pinning centers in MgB_2_ wires with nanoboron grains. The longitudinal section of the wires represented in Figure 2c, Figure 4c, and Figure 7a show that thermal treatment at 740 °C and an isostatic pressure of 1.1 GPa increases the number of connections between grains and allows for the formation of a more homogeneous structure, leading to the improvement of J_ec_ in MgB_2_ wires. When the results for the O sample (nanoboron—740 °C and 1.1 GPa) are compared to the results for undoped MgB_2_ wires [43,44], the HIP process is seen to significantly increase J_ec_ in high magnetic fields.

**Figure 22:**
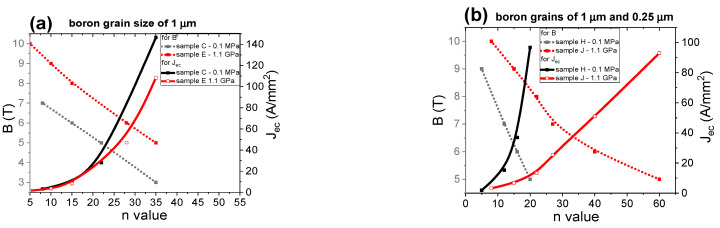

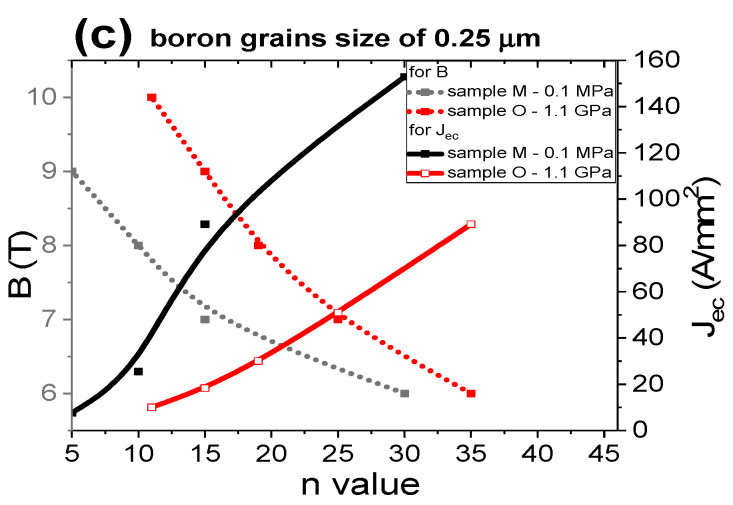
The dependence of the *n* value on the magnetic field (B) perpendicular to the wire axis in undoped MgB_2_ wires for an annealing temperature of 740 °C: (**a**) boron grain size of 1 μm, (**b**) boron grain mixture of 1 μm and 0.25 μm, and (**c**) boron grain size of 0.25 μm.

The results in Figure 22a show that heat treatment under high isostatic pressure significantly increases the *n*-value in MgB_2_ wires with large boron grains. The results in Figure 22a and [27] suggest that MgB_2_ wires with large grain boron after the HIP process can be used to build a medium field superconducting coil, e.g., B = 5 T. These results reveal that the Mg liquid state and an isostatic pressure of 1.1 GPa significantly improve the *n*-value in MgB_2_ wires with large boron grains. The higher *n*-value for sample E is also related to the more homogeneous structure of the MgB_2_ material after the HIP process (Figure 1e and Figure 2c). Further studies show that the HIP process and an annealing temperature of 740 °C lead to a significant improvement in the *n*-value under low, medium, and high magnetic fields in MgB_2_ wires with a mixture of boron grains (Figure 22b). The higher *n*-value for sample J is also related to the more homogeneous structure of the MgB_2_ material after the HIP process (Figure 3e and Figure 4c). The research in Figure 22c shows that thermal treatment under high isostatic pressure significantly increases the *n*-value, especially for high magnetic fields in MgB_2_ wires with nanoboron grains. The results in Figure 22 show that the nanoboron grains most efficiently increase the *n*-value under high magnetic fields. Figure 22 shows that the HIP process and an annealing temperature of 740 °C allow for a high *n*-value under low and medium magnetic fields in MgB_2_ wires with a mixture of boron grains. The results for sample O and the results in [46] (undoped MgB_2_ wires—CTFF method) indicate that the HIP process significantly increases the *n*-value above 7 T. Our research shows that the size of the boron grains significantly influences the critical parameters for the MgB_2_ material during the HIP process, as well as the Mg in the liquid state. Additionally, we proved that the HIP process and Mg in the liquid state improved the homogeneity of the MgB_2_ material structure, leading to improvements in the J_ec_ and *n*-value.

## 4. Conclusions

The results show that a lower filling density of unreacted Mg + B material and the Mg solid-state synthesis reaction lead to a significantly reduced amount of the superconducting phase, a decrease in the homogeneity of the superconducting material, and a significant decrease in the irreversible magnetic field, critical temperature, upper magnetic field, engineered critical current density, and *n*-value, despite the high isostatic pressure treatment and the use of nanoboron in the sample. Our measurements show that samples with large boron grains and an 8% higher filling density of unreacted Mg + B material allow to obtain better critical parameters than MgB_2_ wires with nano boron and a lower filling density of unreacted Mg + B material. Based on these results, we can conclude that the density of the unreacted Mg + B material has a greater influence on Mg solid-state synthesis reactions than boron grain size. The experiments conducted indicate that the density of unreacted material has a negligible impact on B_irr_, T_c_, B_c2_, J_ec_, and the *n*-value for the Mg liquid-state synthesis reaction. In addition, studies show that critical parameters (T_c_, B_irr_, B_c2_, J_ec_, and *n*-value) of MgB_2_ wires depend mainly on the boron grain size for the Mg liquid-state synthesis reaction. Nanoboron grains and the HIP process allow for the highest B_irr_, T_c_, B_c2_, J_ec_, and *n*-value under high magnetic fields. The SEM images taken from the longitudinal section of the wires show that the samples annealed under low isostatic pressure have a highly heterogeneous structure. The HIP process significantly increases the homogeneity of the MgB_2_ material. Transport measurements show that an isostatic pressure of 1.1 GPa and an annealing temperature of 740 °C allow for the highest J_ec_ and *n*-value for MgB_2_ wires with large boron. In addition, the experiments show that the nano boron grains and the solid-state reaction of Mg allow for the formation of the highest number of high-field pinning centers. However, thermal treatment in the liquid state of Mg creates a large number of low- and medium-field pinning centers in MgB_2_ wires with nanoboron. Research has shown that the *n*-value is most decreased in the presence of island structures. The *n*-value has been found to be the best in structures with thin layers and homogeneous layer distributions. The results have shown that layer clustering also results in a reduction in the *n*-value.

## Figures and Tables

**Table 1:** Thermal treatment parameters and initial powder composition of the samples.

No.	Annealing Temperature	Annealing Time	Isostatic Pressure	Boron Grain Size [mm]	Density of the Unreacted Material
	[°C]	[Min]			[G/cm^3^]
A	680	40	0.1 MPa	1	1.5
B	700	40	0.1 MPa	1	1.5
C	740	40	0.1 MPa	1	1.5
D	700	40	1.1 GPa	1	1.5
E	740	40	1.1 GPa	1	1.5
F				50 wt %—1	
	680	40	0.1 MPa	50 wt %—0.25	1.5
G				50 wt %—1	
	700	40	0.1 MPa	50 wt %—0.25	1.5
H				50 wt %—1	
	740	40	0.1 MPa	50 wt %—0.25	1.5
I				50%—1	
	700	40	1.1 GPa	50%—0.25	1.5
J				50%—1	
	740	40	1.1 GPa	50%—0.25	1.5
K	680	40	0.1 MPa	0.25	1.38
L	700	40	0.1 MPa	0.25	1.38
M	740	40	0.1 MPa	0.25	1.38
N	700	40	1.1 GPa	0.25	1.38
O	740	40	1.1 GPa	0.25	1.38

## Data Availability

The data presented in this study are available on reasonable request from the corresponding authors.
